# Characterization of Follicular Atresia Responsive to BPA in Zebrafish by Morphometric Analysis of Follicular Stage Progression

**DOI:** 10.1155/2018/4298195

**Published:** 2018-12-23

**Authors:** M. Migliaccio, T. Chioccarelli, C. Ambrosino, A. Suglia, F. Manfrevola, O. Carnevali, S. Fasano, R. Pierantoni, G. Cobellis

**Affiliations:** ^1^Dipartimento di Medicina Sperimentale, Sez. Bottazzi, Università degli Studi della Campania “L. Vanvitelli”, Via Costantinopoli 16, 80138 Napoli, Italy; ^2^Dipartimento di Scienze e Tecnologie, Università del Sannio, Via Port'Arsa 11, 82100 Benevento, Italy; ^3^Dipartimento Scienze della Vita e dell'Ambiente, Università Politecnica delle Marche, Via Brecce Bianche, 60131 Ancona, Italy

## Abstract

Bisphenol A is an industrial chemical compound, pervasively polluting the environment and diet, classified as an endocrine disruptor because of its interference effects on the endocrine system. In zebrafish, BPA exposure induces follicular atresia. To acquire knowledge on this atretic effect, using a qualitative and quantitative histomorphological approach, we studied zebrafish ovarian follicular stage development in response to low BPA concentrations. Results show that BPA interferes with follicular progression by affecting the previtellogenic and vitellogenic phases. In particular, BPA exposure (i) increases follicular recruitment by acting on primary stage follicles, (ii) forces the follicular transition from stage III to stage IV producing enlarged stage IV follicles, and (iii) induces atresia by producing atretic follicles that are peculiarly enlarged (i.e., big atretic follicles). We suggest that BPA induces atresia by the primary effect on recruitment of stage I follicles. This forces follicular progression and produces stage IV follicles that are peculiarly enlarged that undertake the atretic development.

## 1. Introduction

Bisphenol A (BPA) is one of the highest volume chemicals produced worldwide. It is used to manufacture plastics, epoxy resins, hard plastic bottles, and metal-based food and beverage cans. It is a pervasive industrial chemical compound, broadly present in the environment and diet [1]; hence, it is not surprising that over 90% of the tested humans have detectable level of BPA in biological fluids, with the highest levels reached in infants and children [2–5]. In contrast to toxicokinetic studies of human BPA metabolism, available data from biomonitoring studies indicate the presence of measurable concentrations of BPA in individual adults, adolescents, and children [5], as well as in the maternal/fetal circulation, amniotic fluid, and placental tissue of pregnant women [6] demonstrating that the human population is exposed to BPA and that the placenta does not act as a barrier.

BPA is one of the endocrine disruptors (EDCs) whose (anti)estrogenic and antiandrogenic activity, widely documented in the past, was exerted by nuclear and extranuclear mechanisms [7, 8]. This observation is the base of the growing concern on BPA because it interferes with hormonal signaling pathways even at very low doses. Recently, its action on the signaling pathway other than the nuclear receptor has been reported mainly due to its ability to promote oxidative stress response pathways [9] and nuclear factor kappa-light-chain-enhancer of activated B cells (NF-*κ*B), among the others deeply impairing the transcriptional activity [10, 11]. Epidemiological and experimental studies reveal the relationships between BPA exposure and several diseases, including reproductive dysfunctions. Notably, BPA impairs fertility, in both male and female, by activating or antagonizing sex nuclear receptors in target reproductive tissues [1, 12–16], including the estrogen (i.e., ER*α*, ER*β*, and GPR30) and androgen receptors [17–20]. In this context, BPA exposure results in being a power risk factor for infertility.

In mammals, the ovary is sensitive to disruption by EDCs, especially when exposure occurs during intrauterine [21–23] or early postnatal life [24]. In particular, there is growing evidence that BPA has the potential to impact at different stages of oocyte development; in fact, *in utero* exposure to low doses of BPA disrupts early oogenesis in mice and in rhesus monkey [25, 26], while in the neonatal mouse ovary, BPA promotes the transition of primordial follicles to primary follicles and suppresses the meiotic maturation of oocytes due to abnormal spindle assembly during meiosis I [27, 28]. Interestingly, these effects of BPA on the growing oocytes are modulated by phytoestrogens in the diet [29]. In rats, BPA exposure during the early postnatal period decreases the primordial follicle reserve [30] and alters ovarian morphology leading to a large number of cysts and infertility [31]. In agreement, the neonatal exposure to low doses of BPA in lambs decreased the proportion of primordial follicles (with acceleration of the follicular recruitment) and ovarian reserve and increased the number of atretic follicles [24].

However, the published reports on the occurrence and/or severity of the follicular effects of BPA show contrasting data, presumably reflecting differences in the experimental approaches (e.g., the use of different species or strains of animals or the doses, duration, route, or timing of the exposures).

Since the aquatic environment is a major sink for EDCs and fishes are particularly vulnerable to exposure to EDCs, the zebrafish (*D. rerio*) has recently emerged as an important tool for monitoring water pollution [32, 33] and for studying their ecotoxicology and biological effects, including the reproductive ones [34–38]. In zebrafish, BPA exposure affects ovarian follicles [15, 39, 40]. This chemical triggers alterations in follicular development, causing an increase in the percentage of atretic follicles, as the exposure concentration rises [39, 40]. In this context, we selected zebrafish as an animal model to better assess the reproductive effects of BPA and, in collaboration with Carnevali and coworkers, we recently characterized the effects of BPA on the zebrafish ovary at the phenotypic and molecular level. We showed that the exposure to low BPA concentrations (5 *μ*g/L, similar to environmental concentration) promotes follicular atresia and anovulation. These effects were not observed at higher BPA concentrations (10 and 20 *μ*g/L). At a molecular level, we showed that 5 *μ*g/L BPA promotes no transcriptional effect on genes involved in steroidogenesis (estrogen receptor, *esr2b*; steroidogenic acute regulatory protein, *star*; and cytochrome P450 family 11 subfamily A member, *cyp11a1*) and oocyte growth (follicle-stimulating hormone receptor, *fshr*) but significantly downregulates the expression of genes involved in ovarian development (estrogen receptors, *esr1* and *esr2a*) [15], production of maturation hormone (luteinizing hormone/choriogonadotropin receptor, *lhcgr*), and acquisition of maturational competence (progesterone receptor membrane component, *pgrmc1* and *pgrmc2*) with an increase in apoptotic gene expression (*caspase3* and *tumor protein 53*).

In this context, to acquire knowledge on the 5 *μ*g/L BPA effects inducing follicular atresia, using a qualitative and quantitative histomorphological approach, we evaluated ovarian follicle stages in response to BPA exposure. In particular, using the ovaries collected and molecularly already analyzed by Carnevali and coworkers [15], we morphometrically characterized the area of atretic follicles, as well as the number, progression, and growth of the follicular stages by histological analysis. The aim was to characterize the BPA-induced atresia (atretic effects) and decode the follicular stage primarily targeted by BPA exposure.

## 2. Material and Methods

### 2.1. Animals and BPA Exposure

Adult female zebrafish (*D. rerio*, AB strain, wild-type background) have been exposed to BPA by Carnevali and coworkers (see [15]). As already reported, 48 females were divided in eight 10 L aquaria with oxygenated water under controlled conditions (28.0 ± 0.5°C), maintained on a 14/10 h light/dark cycle, and fed 4 times a day, twice commercial food (Vipagran; Sera, Loessnitz, Germany) and twice *Artemia salina*. In particular, 48 females were divided into 4 experimental groups. Each group was divided in two tanks, each containing 6 fishes, in order to obtain biological replicates. There were 1 control and 3 exposed groups, which received 5, 10, or 20 *μ*g/L BPA (98% analytical purity, Sigma-Aldrich, Milano, Italy) for 3 weeks. Each experimental group contained 12 fish. This number has been fixed in excess by G^∗^Power analysis using G^∗^Power 3.1.9.2 software which suggested a total of 40 animals (10 animals/group) to have 0.82 as power calculation, with *p* value fixed to 0.05.

The BPA was diluted in absolute ethanol (EtOH), used as a vehicle, and added to water (final dilution < 1 : 10000). Since the extensive dilution, the control group was reared only in fresh water without EtOH, according to the Organization for Economic Cooperation Development for chronic assays (OECD 2012). Furthermore, since pilot studies have shown that the bioavailability of BPA was reduced by 50% after 4 days, the treatment with BPA was performed by renewing the water tank with the diluted pollutant (5 *μ*g/L BPA) every three days.

After 3 weeks of treatment, animals were lethally anesthetized with 500 mg/L MS-222 (3-aminobenzoic acid ethyl ester, Sigma-Aldrich) buffered to pH 7.4. For histological analyses, ovaries (*n* = 10 half ovaries/experimental group) were fixed in Bouin's solution.

All procedures complied with the Italian animal experimentation laws and were approved by the Ethics Committee of Padua University (Prot. 112/2015-PR).

### 2.2. Tissue Histological Processing

In a previous work carried out in collaboration with Carnevali and coworkers, it has been reported that zebrafish exposed to the lowest BPA concentration (5 *μ*g/L) showed blocked ovulation by atresia. The intermediate (10 *μ*g/L) and the highest (20 *μ*g/L) BPA concentration did not induce significant changes with respect to the control group.

Moving from these results and using the same ovaries collected and molecularly analyzed in the previous study [15], here we further characterized the reported atresia by the analysis of follicle development using a morphometric approach.

Ovaries from CTRL and BPA-exposed (5 *μ*g/L) zebrafish were processed for histological examination as already reported [41]. Gonads (*n* = 10 half ovaries CTRL and 10 half ovaries BPA) were embedded in paraffin, sectioned (7 *μ*m) with a microtome, processed for hematoxylin-eosin staining (using standard procedures), and observed at 100x final magnification (×10 objective, ×10 ocular) under a light microscope (Leica Microsystems Inc., Milano, Italy). Images were captured using a high-resolution digital camera (DC300F; Leica Microsystems Inc., Milan, Italy), and, in combination with the direct observation at the microscope, these were used to analyze follicular stages. In particular, 3 serial sections out of every 10 sections (70 *μ*m interval between the analyzed sections, at least 12 total sections/experimental group) were used to identify and count follicles, as well as to analyze the follicular area size. In BPA-exposed ovaries, the follicles appeared fragile to sectioning; therefore, the analyses were carried out using 10 CTRL and 9 BPA-exposed ovaries.

As routinely required for these experimental procedures, statistical analysis was carried out using the mean values of 3 serial sections; also, all the analyses were validated using double-blind testing by two observers.

### 2.3. Follicular Stage Identification

In accordance with Selman et al. [42], we identified and classified five stages of follicular development. In particular, the primary follicle (primary growth stage I) was identifiable by few peripherally located nucleoli as well as by intense basophilia of the cytoplasm. It is noteworthy that at this stage, the follicle starts to form and the oocyte increases and progresses through the early stages of meiosis I which will then stops temporarily in prophase I.

The cortical alveolar stage II was identifiable by the appearance of cortical alveoli (i.e., “yolk” vesicles containing zona pellucida proteins) accumulating within the oocytes. The nucleus appeared structurally regular and enlarged and contained multiple peripheral nucleoli.

During vitellogenic stages, the follicles increased in size due to the accumulation of the yolk, and the zona radiata was clearly evident. The irregular structure of the nucleus was accompanied by invaginations of the nuclear envelope. At the appearance of yolk granules, the follicles were classified as early vitellogenic stage III (i.e., maturational incompetent). The follicles were classified as late vitellogenic and maturational competent stage IV when yolk granules became abundant and started to merge and the peripheral migration of the germinal vesicle was observed. In this stage, germinal vesicle breakdown occurs (i.e., final meiotic maturation).

Mature or spawning stage V was identified by the absence of the germinal vesicle (i.e., nuclear membrane).

As previously reported [15], the identification of atretic follicles (A) was based on atretic morphological markers characterized by Üçüncü and Çakıcı [43] such as granulosa cell hyperplasia, thinning, invagination and breakdowns of the zona radiata, basal membrane disintegration, and absorption of vitellus.

### 2.4. Follicular Stage Count

For each ovary, a minimum of 4 random sections (each of these included 3 serial sections out of every 10 sections at least) were used to identify and count follicles (i.e., total and specific follicular stages).

Follicular stage I (primary growth), stage II (cortical alveolar), stage III (early vitellogenic), stage IV (late vitellogenic and maturational competent), and stage V (mature) were classified and counted in both experimental groups. Using the morphological mark of atresia, we also counted the atretic follicles (hence forwardly referred to as atretic).

The number of follicles in each stage relative to the total follicles (i.e., percentage of follicular stages) has been analyzed and graphed as mean values of 3 serial sections ± SEM.

The number of stage III and stage IV relative to the total vitellogenic follicles (i.e., percentage of stages III-IV/total vitellogenic stages) has been analyzed and graphed. In particular, total vitellogenic follicles have been counted and classified in early (stage III) and late (stage IV) vitellogenic stages. The percentage of follicles at stages III and IV (number of stages III and IV/total vitellogenic follicles) has been graphed as mean values of 3 serial sections ± SEM.

### 2.5. Analysis of Follicular Size

The follicular size of vitellogenic, mature, and atretic follicles from control and BPA-exposed zebrafish was analyzed by evaluating the follicular area using ImageJ software. For each ovary, a minimum of 4 random sections were evaluated and the mean area for each follicle was evaluated on 3 serial sections. Results have been expressed in square pixels (p^2^) and graphed as mean values of 3 serial sections ± SEM.

The mean area of atretic follicles (1.9 p^2^) from control ovaries was used as reference values. In both experimental groups, the atretic follicles with lower and upper reference area value were graphically plotted and statistically analyzed by Fisher's chi-squared (*χ*^2^) test. The larger atretic follicles (i.e., >1.9 p^2^) have been referred to as big atretic follicles.

### 2.6. Statistical Analysis

Student's *t*-test or ANOVA followed by Duncan's test for multigroup comparison was performed to evaluate the significance of differences, as a proper. Data were expressed as the mean of the serial sections ± SEM. *p* < 0.05 and 0.01 were considered statistically significant.

Fisher's *χ*^2^ test, with 1 degree of freedom, was performed using Microsoft Excel for testing relationships between the two variables, i.e., the treatment and area of atretic follicles. The critical value of *χ*^2^ has been considered significant when it has a lower probability level (i.e., *p* < 0.01).

## 3. Results and Discussion

In collaboration with Carnevali and coworkers, we have already reported that the exposure to low-dose BPA (5 *μ*g/L BPA) promoted follicular atresia interfering with the expression of genes involved in the development and maturation of the zebrafish ovary. Moving from these results and using the same ovaries collected and molecularly analyzed in the previous study [15], we characterized the atresia by the analysis of follicle development using a morphometric approach.

Ovarian sections from control and 5 *μ*g/L BPA-exposed zebrafish (BPA-zebrafish) were processed for H&E staining and used to characterize, either morphologically or morphometrically, the area of atretic follicles in response to BPA.

**Figure 1:**
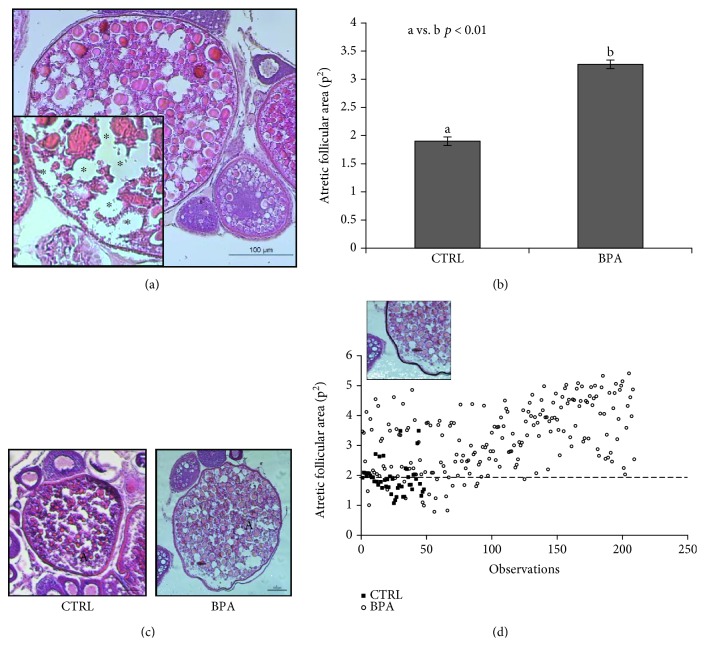
(a) Ovary section from zebrafish exposed to BPA (5 *μ*g/L) stained with hematoxylin-eosin dye (H&E). The atretic follicle is shown. The inset shows morphological markers of atresia. (b) The atretic follicle area from unexposed (CTRL) or exposed zebrafish to BPA (5 *μ*g/L). Results are representative of four animals/group analyzed separately and expressed as mean area ± SEM (p^2^). a vs. b *p* < 0.01. (c) Atretic follicle from unexposed (CTRL) and exposed zebrafish to BPA (5 *μ*g/L) stained with H&E. (d) Distribution diagram of atretic follicle area values (p^2^) from CTRL (filled square) and BPA-exposed (open circle) zebrafish. The dotted line represents the mean area of atretic follicles from the CTRL group. The figure shows black line outlined using the ImageJ tool for the evaluation of the follicular area. Scale bar: 100 *μ*m, ∗: empty area; A: atretic follicle.

We observed that BPA modified the morphology and area of the atretic follicles. Most of these follicles appeared enlarged and fragile to sectioning and contained empty areas (i.e., histologically identifiable as white areas) peripherally localized or randomly spread (Figure 1(a)). The morphometric analysis demonstrated that BPA significantly (*p* < 0.01) increased the mean area of atretic follicles (3.27 ± 0.08 p^2^) compared to that of the control group (1.9 ± 0.08 p^2^) (Figure 1(b)). Specifically, BPA exposure produced atretic follicles that are markedly large (big atretic follicles) (Figure 1(c)), suggesting a follicular atresia peculiarly associated with BPA exposure. To evaluate numerically our observations, we considered the mean area of control atretic follicles (1.9 p^2^) as the reference value and counted atretic follicles with a higher reference area value (i.e., >1.9 p^2^ corresponding to big atretic follicles) (Figure 1(d)). Only a few number of big atretic follicles (min-max area values ranging from 1.92 to 3.49 p^2^) were present in the control group. On the contrary, a significant increase in the number of big atretic follicles (min-max area values ranging from 1.91 to 5.41 p^2^) was present in BPA-exposed animals. Statistical analysis (*χ*^2^ = 0.00003) demonstrated a significant higher incidence of “big atretic follicles” in BPA-exposed ovaries (CTRL vs. BPA, *p* < 0.01). The results suggest that BPA induced a follicular atresia characterized by “big-sized” follicles.

In order to study the follicular swelling characterizing the BPA-induced atresia (BPA-atresia), we attempt to identify the follicular stage primarily targeted by the exposure. In particular, we studied the effects of BPA on previtellogenic and vitellogenic phases of follicular development. Ovarian sections from control and BPA-zebrafish were processed for H&E staining and used to analyze the morphology, number, growth, and progression of the follicular stages using a qualitative and quantitative histomorphometrical approach.

**Figure 2:**
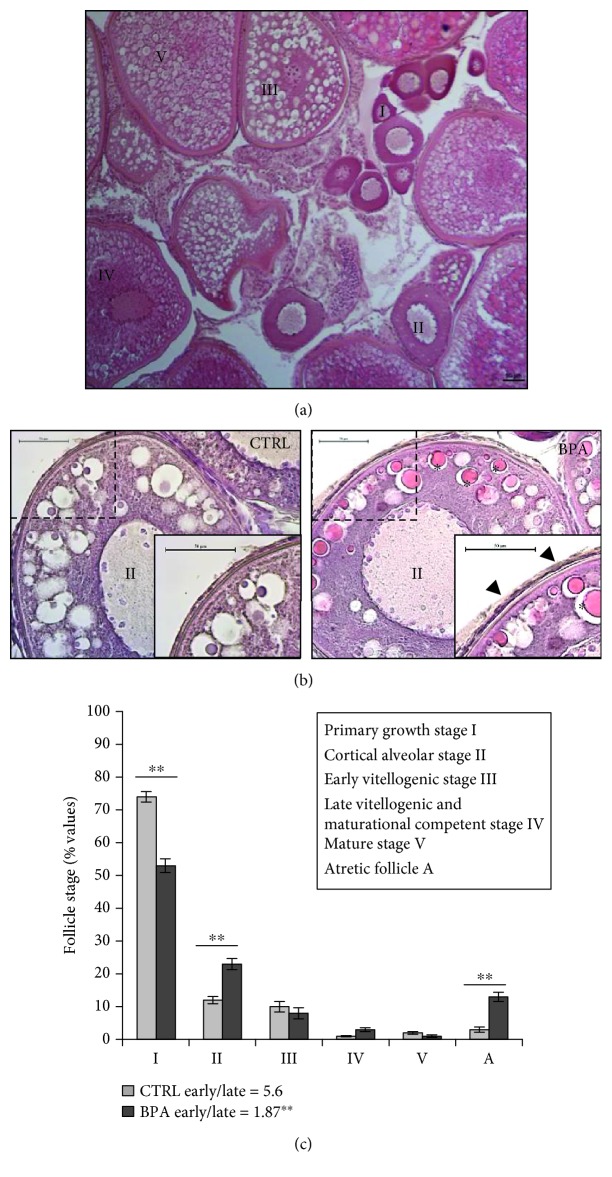
(a) Ovary section from zebrafish stained with H&E. Different follicular stages are shown. Scale bar: 50 *μ*m. (b) Cortical alveolar stage II follicle from CTRL and BPA-exposed ovarian zebrafish stained with H&E. The inset is the enlarged view of the dashed line square. Scale bar: 50 *μ*m. Asterisks (∗) indicate yolk granules while arrows indicate the zona pellucida envelope which is intensely evident. (c) Number of follicles in each developmental stage from CTRL and BPA-exposed ovarian zebrafish. Results are representative of four animals/group analyzed separately and expressed in percentage (number of follicles in each stage/total follicles) as mean ± SEM. Early/late vitellogenic follicle ratio is reported for both experimental groups. Asterisks indicate statistically significant differences (^∗∗^*p* < 0.01). I: primary growth stage I; II: cortical alveolar stage II; III: early vitellogenic stage III; IV: late vitellogenic stage IV; V: mature stage V; A: atretic follicle.

In agreement with our previous study [15], the histomorphological analysis of tissues showed that all the follicular stages, from primary to mature stages, were present in both experimental groups (Figure 2(a)). Apparently, in BPA-exposed ovaries (BPA-ovaries), no morphological change was observed with respect to the control group except for the atresia, as described above, and the presence of many stage II follicles characterized by morphological markers generally ascribable to stage III vitellogenic follicles. Indeed, we noted the presence of cortical alveolar follicles with premature formation and of zona pellucida (ZP) envelope and yolk granules. Both were intensely evident and acidophil (see CTRL vs. BPA in Figure 2(b)) suggesting that BPA prematurely forced the follicular growth phase. Similar to our observation, ovaries from zebrafish exposed to higher BPA dose (10 *μ*g/L) showed cortical alveolar follicles with early formation of the zona pellucida [39]. Interestingly, one of the most important reported responses to estradiol-17*β* and environmental estrogens is the induction of vitelline envelope *zp* proteins [44, 45], suggesting that, in our experimental model, BPA affected the follicular growth by interfering with estrogenic signaling. Accordingly, molecular data previously reported using ovaries from the same animal groups analysed herein [15] showed that BPA exposure downregulated the expression of both estrogen receptors (*esr1* and *esr2a*) whereas it did not modify the steroidogenic gene transcription.

Growing evidences demonstrate that BPA affects the early follicular growth and numerically increases the atretic follicles [24–26, 29, 39, 46]. In zebrafish [39, 46], as well as in different mammalian species [24, 30], BPA decreases the number of primordial follicles and induces atresia. In both mouse and lamb [24, 25], this effect has been related to the acceleration of the follicular recruitment. In agreement with these findings, counting of follicular stages in control and BPA-zebrafish (Figure 2(c)) revealed that the number of stage I follicles was significantly lower in BPA-ovaries compared to the control group (*p* < 0.05), whereas the number of stage II follicles significantly increased (*p* < 0.05). Surprisingly, we observed no effect on the number of early (stage III) and late (stage IV) vitellogenic follicles, nor on the mature ones. On the contrary, the ratio between early and late vitellogenic follicles apparently decreased after BPA exposure, and the atretic follicles were numerically higher (CTRL vs. BPA, *p* < 0.01). This suggested the BPA ability to promote atresia by forcing the vitellogenic phase.

To verify this hypothesis, we investigated the BPA effects on stage III-IV transition by counting the number of stage III and IV follicles relatively to total vitellogenic ones.

**Figure 3:**
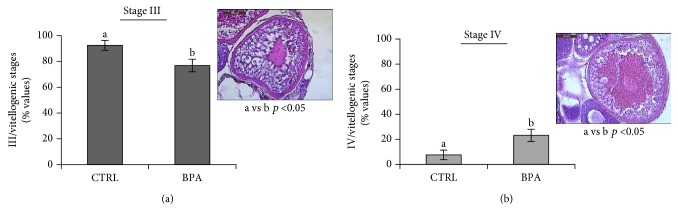
Number of stage III (a) and stage IV (b) relative to the total vitellogenic follicles from CTRL and BPA-exposed ovarian zebrafish. Results are representative of four animals/group analysed separately and expressed in percentage (number of stage III or IV/total vitellogenic follicles) as mean ± SEM. Letters indicate statistically significant differences, a vs. b *p* < 0.05. The images show (a) early vitellogenic stage III and (b) late vitellogenic stage IV follicles stained with H&E. Scale bar: 100 *μ*m.

The results reported in Figures 3(a) and 3(b) point out that BPA exposure significantly decreased (*p* < 0.05) the percentage of early vitellogenic stage III vs. the control group (Figure 3(a)). Conversely, the percentage of late vitellogenic stage IV significantly increased (*p* < 0.05) in BPA-exposed animals (Figure 3(b)), confirming the hypothesis that BPA accelerated the progression of the vitellogenic phase.

Altogether, the above-reported results demonstrate that BPA exposure elicited a double effect, particularly on previtellogenic (follicular recruitment and growth) and vitellogenic phases, suggesting potential impacting effects on ovarian reserve and atresia. Indeed, BPA forced the transition from stage I to stage II as well as from stage III to stage IV (i.e., early to late) and significantly increased the number of “big atretic follicles.” The high incidence of stage II follicles suggested further interference on follicular development. We believe that the premature development of stage II follicles slowed down their vitellogenic entry by interfering with the transition from previtellogenic to vitellogenic phase. Likely, this occurred because of the transcriptional downregulation of estrogen receptors (*esr1a* and *esr2a*) previously reported in our experimental model [15]. In agreement, a few transcriptional decrease in *esr2a* was observed in ovaries of *Gobiocypris rarus* exposed to BPA (50 *μ*g/L for 5 weeks) and was associated with the exclusive presence of previtellogenic and atretic follicles [47]. More importantly, the genetic loss of estrogen receptors *esr2a/b* in zebrafish led to an arrest of folliculogenesis at previtellogenic stage II [48] demonstrating a role for esr2 in follicular growth. Accordingly, whereas *esr1* and *esr2b* are expressed in the zebrafish follicle, exclusively in somatic cells, the *esr2a* gene is responsive to BPA and is expressed in both somatic and germinal cells, and it is the most abundantly expressed form during follicle growth [49]. In zebrafish, oocytes at early stage III are known to be maturational incompetent. During the vitellogenic phase, stage III follicles undergo vitellogenesis resulting in an increased follicular size. It has been reported that the large late stage IV oocytes show an increased expression of membrane progesterone receptor (*pgrmc*) and acquire responsiveness to LHCGR-stimulated maturation thus developing into mature follicles. The molecular data previously reported, using ovaries collected from the same animal groups analysed herein [15], indicated that BPA exposure inhibited the expression of genes regulating oocyte maturation (i.e., *lhcgr*, *pgrmc*, and *esr1*). The data evidenced that BPA exposure promoted follicular atresia interfering with the maturational phase.

Here, we report that BPA increased follicular recruitment by acting on stage I-II transition and forced the vitellogenic phase, from early stage III to late stage IV, without affecting the number of the mature follicles, implying that BPA produced “big atretic follicles” independently of classical follicular atresia mechanisms.

Considering these aspects, we hypothesized that BPA might promote follicular swelling characterizing the atresia by affecting vitellogenic growth stages more than the mature ones. This hypothesis was verified evaluating the potential “swelling effect” of BPA on vitellogenic and maturational follicles by the analysis of the follicular area of stages III, IV, and V in control and BPA-ovaries.

**Figure 4:**
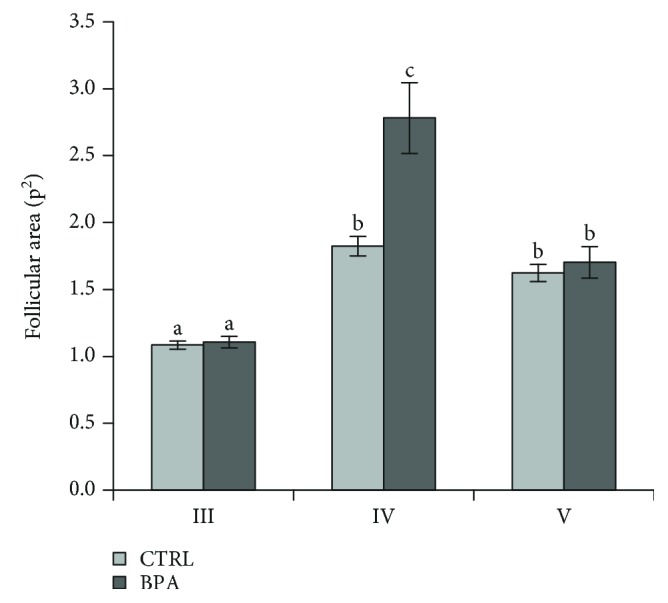
Stage III to V follicle area from CTRL and BPA-exposed ovarian zebrafish. Results are representative of four animals/group analyzed separately and expressed as mean area ± SEM (p^2^). a vs. b *p* < 0.01; a vs. c *p* < 0.01; b vs. c *p* < 0.01. III: early vitellogenic stage; IV: late vitellogenic maturational competent stage; V: mature stage.

In both experimental groups, the follicular area of stage IV (Figure 4) significantly increased in comparison to the area of stage III (*p* < 0.01). The efficiency of such increase was significantly higher in BPA-ovaries than in control groups (*p* < 0.01) demonstrating that BPA promoted follicular swelling during the vitellogenic phase. However, the follicular area of stage V was comparable to that of control stage IV indicating that the maturation physiologically occurred. Indeed, no mature follicle with abnormal size was observed in control or exposed ovaries.

Although further studies will be necessary to define if the described effects by BPA are transient or permanent, the results reported herein suggest that BPA exposure primarily affects early follicular stages. We show that BPA forces the recruitment of stage I follicles as well as the follicular transition from stage III to stage IV with the production of stage IV follicles that are abnormally enlarged and big atretic follicles. We suggest that BPA forces the early follicular recruitment and peculiarly induces atresia by pushing follicular growth mechanisms associated with the vitellogenic phase. Interestingly, it has been reported that BPA interferes with the transcriptional expression of vitellogenin and cell-to-cell junctional proteins [47, 50] which are reported to be key proteins of vitellogenesis [51].

## 4. Conclusions

The morphological observations reported here demonstrate that BPA interferes with follicular progression affecting the previtellogenic and vitellogenic phases. In particular, BPA exposure (i) interferes with the recruitment of stage I follicles by pressing stage I to stage II transition, (ii) forces the follicular transition from stage III to stage IV, (iii) slows down follicular progression from the previtellogenic to vitellogenic phase, (iv) enlarges late vitellogenic stage IV follicles, and (v) induces atresia by producing atretic follicles that are peculiarly enlarged (i.e., big atretic follicles).

**Figure 5:**
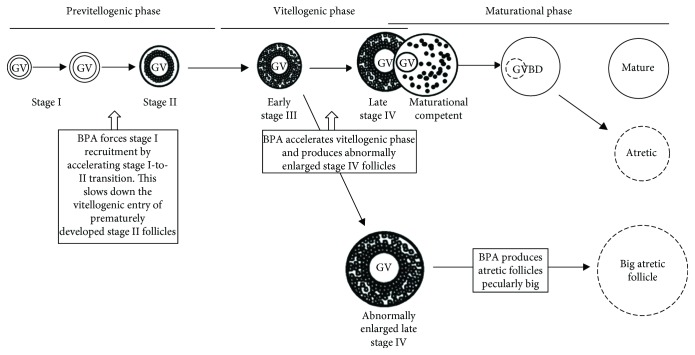
Schematic representation of BPA effects on the zebrafish follicular development and hypothetical model of follicular atresia, peculiarly responsive to BPA. The exposure to low doses (5 *μ*g/L) of BPA promotes the atretic development of the early recruited stage I follicles by accelerating and forcing their progression throughout previtellogenic and vitellogenic phases. The exposure produces stage IV follicles abnormally enlarged thus pressing their peculiar atretic development. Scheme adapted from Clelland et al. (2009).

We suggest that BPA promotes atresia by impairing follicular recruitment. We propose a potential model of follicle atresia peculiarly responsive to BPA (Figure 5) in which BPA promotes the atretic development of the early recruited stage I follicles by accelerating and forcing their progression throughout previtellogenic (i.e., fast stage I-to-II transition) and vitellogenic (i.e., fast stage III-to-IV transition; abnormally enlarged stage IV) phases. We believe that the premature development of stage II follicles (see the premature appearance of ZP and yolk granules) slows down their vitellogenic entry. This effect of BPA on follicular development implicates that BPA promotes the production of big atretic follicles independently of classical mechanism of follicular atresia, responsive to oocyte maturation failure. This might explain why, in our experimental model, BPA exposure does not affect the number of the mature follicles.

Of course, further analysis needs to decode clearly the molecular mechanisms promoting atresia responsive to BPA, although our data unequivocally show that ovary zebrafish is sufficiently sensitive, to verify the effects of environmental concentration of BPA by simple histomorphological evaluation of late vitellogenic and atretic follicular size.

## Data Availability

The data used to support the findings of this study are available from the corresponding author upon request.
